# Genes *CEP55*, *FOXD3*, *FOXF2*, *GNAO1*, *GRIA4*, and *KCNA5* as potential diagnostic biomarkers in colorectal cancer

**DOI:** 10.1186/s12920-019-0501-z

**Published:** 2019-04-15

**Authors:** Nina Hauptman, Daša Jevšinek Skok, Elena Spasovska, Emanuela Boštjančič, Damjan Glavač

**Affiliations:** 1 Institute of Pathology, Faculty of Medicine, University of Ljubljana, Korytkova 2, SI-1000 Ljubljana, Slovenia; 2 Agricultural Institute of Slovenia, Hacquetova ulica 17, SI-1000 Ljubljana, Slovenia

**Keywords:** Colorectal cancer, Methylation, Expression, Bioinformatics approach, Experimental validation

## Abstract

**Background:**

Colorectal cancer (CRC) is one of the leading causes of death by cancer worldwide and in need of novel potential diagnostic biomarkers for early discovery.

**Methods:**

We conducted a two-step study. We first employed bioinformatics on data from The Cancer Genome Atlas to obtain potential biomarkers and then experimentally validated some of them on our clinical samples. Our aim was to find a methylation alteration common to all clusters, with the potential of becoming a diagnostic biomarker in CRC.

**Results:**

Unsupervised clustering of methylation data resulted in four clusters, none of which had a known common genetic or epigenetic event, such as mutations or methylation. The intersect among clusters and regulatory regions resulted in 590 aberrantly methylated probes, belonging to 198 differentially expressed genes. After performing pathway and functional analysis on differentially expressed genes, we selected six genes: *CEP55*, *FOXD3*, *FOXF2*, *GNAO1*, *GRIA4* and *KCNA5*, for further experimental validation on our own clinical samples. In silico analysis demonstrated that *CEP55* was hypomethylated in 98.7% and up-regulated in 95.0% of samples. Genes *FOXD3*, *FOXF2*, *GNAO1*, *GRIA4* and *KCNA5* were hypermethylated in 97.9, 81.1, 80.3, 98.4 and 94.0%, and down-regulated in 98.3, 98.9, 98.1, 98.1 and 98.6% of samples, respectively. Our experimental data show *CEP55* was hypomethylated in 97.3% of samples and down-regulated in all samples, while *FOXD3*, *FOXF2*, *GNAO1*, *GRIA4* and *KCNA5* were hypermethylated in 100.0, 90.2, 100.0, 99.1 and 100.0%, and down-regulated in 68.0, 76.0, 96.0, 95.2 and 84.0% of samples, respectively. Results of in silico and our experimental analyses showed that more than 97% of samples had at least four methylation markers altered.

**Conclusions:**

Using bioinformatics followed by experimental validation, we identified a set of six genes that were differentially expressed in CRC compared to normal mucosa and whose expression seems to be methylation dependent. Moreover, all of these six genes were common in all methylation clusters and mutation statuses of CRC and as such are believed to be an early event in human CRC carcinogenesis and to represent potential CRC biomarkers.

**Electronic supplementary material:**

The online version of this article (10.1186/s12920-019-0501-z) contains supplementary material, which is available to authorized users.

## Background

Colorectal cancer (CRC) is one of the leading causes of death by cancer in both genders [1]. CRC occurs through a process of malignant transformation, when numerous genetic and epigenetic events transform normal colon mucosa to adenocarcinoma [2]. It is a very heterogeneous disease, in which three major molecular pathways have been identified. The most common pathway is the chromosomal instability (CIN) pathway, which is characterized by an accumulation of mutations in specific genes (e.g., *APC*, *KRAS*, *BRAF*, *TP53*) [2], and accounts for 65–70% of sporadic CRC cases [3]. The microsatellite instability (MSI) pathway accounts for approximately 15% of sporadic CRC, and is characterized by deficiency in DNA mismatch repair (MMR) genes (e.g. *MLH1*, *MSH2*, *MSH6*, *PMS2*) [4]. Silencing of MMR genes in the MSI type of CRC occurs through promoter hypermethylation, a common molecular alteration at epigenetic level. In more than 80% of MSI cases, promoter hypermethylation occurred in the *MLH1* gene [5]. The third molecular pathway is the CpG island methylator phenotype (CIMP); an epigenetic instability pathway. One of these three pathways is usually predominant but they are not mutually exclusive [6, 7].

CIMP has been extensively studied, not only in CRC but also in bladder, gastric, lung and breast cancer [8]. Some researchers have proposed three CIMP subtypes: CIMP high (CIMP-H), CIMP low (CIMP-L), and non-CIMP subtypes [5]. The CIMP-H subtype is significantly associated with the proximal colon and mutations in gene *BRAF*, whereas the CIMP-L subtype has intermediate methylation levels and is associated with mutations in KRAS gene [9]. Moreover, The Cancer Genome Atlas (TCGA) consortium describes four epigenetic subtypes (CIMP-H, CIMP-L, Cluster 3, and Cluster 4), of which Clusters 3 and 4 are defined as non-CIMP subtypes [10].

Whereas two research groups, Shen et al [11] and Yagi et al [12], reported three epigenetic subtypes and some genes as hypermethylation markers, Hinoue et al. identified four subtypes based on hierarchical clustering of DNA methylation at loci exhibiting high inter-tumor variability [13]. Two loci, representing CIMP-H and CIMP-L tumors, were associated with *BRAF* and *KRAS* mutations, respectively. Tumors in the third cluster were associated with *TP53* mutations and prevalence in the distal colon, while the fourth cluster was enriched for tumors from the rectum, with low rates of *KRAS* and *TP53* mutations. Moreover, previous studies have suggested that differences in the CIMP status are associated with differences in the transcriptomic level across several tumor types [8].

Using bioinformatics approach to select and validate markers aberrantly methylated in CRC has been attempted many times. Integration of epigenomics and genomics data identified 27 genes with hypermethylation/down-regulation, of which *ADHFE1*, *BOLL*, *SLC6A15* and *TFPI2, and TFPI2, EYA4, NPY, TWIST1, LAMA1 and GAS7 were experimentally validated* [14, 15]*. Another study suggested 8 genes, ADHFE1*, *C1orf70*, *SND1*, *OPLAH*, *TLX2*, *ZFP64*, *NR5A2* and *COL4A with diagnostic potential in CRC* [16]*.*

*Our aim was also to identify new aberrantly methylated gene promoters and observe their expression. Our approach however was different. We* used the data from TCGA, in which the DNA methylation experiment was done using microarrays, containing over 450.000 sites within the genome. Unsupervised clustering of methylation data resulted in four clusters and each was compared to the methylation data of normal mucosa samples. The aberrantly methylated probes were intersected among all clusters to obtain the probes common to all clusters. The common methylation sites in all clusters were integrated with gene expression analysis, to identify novel candidate biomarkers, some of which we tested on our experimental set of samples. Finding common epigenetic alterations in all CRC types, regardless of tumor stage, could be a starting point for testing these methylation changes on cfDNA obtained from patient’s blood and/or novel therapeutic targets.

## Methods

### Bioinformatics methods

#### Patients and data

Colon adenocarcinoma (COAD) and rectum adenocarcinoma (READ) data were obtained from The Cancer Genome Atlas (TCGA). Data were downloaded from the Broad GDAC Firehose portal (https://gdac.broadinstitute.org/) and contained clinical information, methylation, gene expression and mutation data. Platform used for DNA methylation experiment was Illumina Infinium HumanMethylation450k BeadChip array (HM450), which covers 482,421 CpG sites within the human genome. For methylation analysis level 3 data was used, which is already normalized and contains beta-value calculations, genomic coordinate, chromosome number and HUGO gene symbol for each CpG site on the array. For gene expression analysis mRNAseq experiment performed on Illumina HiSeq platform was used. Gene expression levels were obtained through RNAseqV2 pipeline, which uses a combination of MApSplice and “scaled estimate” (RSEM) to determine expression levels. RNAseqV2 data contains a normalized read count, which represents normalized RSEM count estimates from the upper quartile. Mutation data was obtained through variant calling from DNAseq experiment using MuTect2 pipeline. There were 381 tumor samples with methylation data on HM450 platform and complete mutational profile. From these 381 samples, 359 samples had also Illumina mRNAseqV2 gene expression data. There were 45 normal samples used for comparison in methylation data and 51 normal samples used for comparison in the gene expression dataset.

#### Probes and genes

The coordinates of protein-coding genes were downloaded from Ensembl, release 89 (http://www.ensembl.org/). The nomenclature of genes was unified according to The HUGO Gene Nomenclature Committee (HGNC) (http://www.genenames.org/). We mapped the HM450 probes to the GRCh38/hg38 genome using recently published study [17]. Location of mapped probes were overlapped them with promoter regions of regulatory build of genome and assigned to their nearest genes. The genes where transcription start site was within 5 kb of the mapped promoter region were used for further analysis.

#### Unsupervised clustering

We used the recursively petitioned mixture model (RPMM) for the identification of colorectal tumor subgroups based on the HM450 DNA methylation data. RPMM is a model-based unsupervised clustering approach developed for beta-distributed DNA methylation measurements that lie between 0 and 1 and is implemented as the RPMM Bioconductor package [18]. We removed probes mapped on X and Y chromosome and the probes containing “NA” values and performed RPMM clustering on 4165 probes, that showed the most variable DNA methylation levels (standard deviation > 0.25). A fanny algorithm (a fuzzy clustering algorithm) was used for initialization and level-weighted version of Bayesian information criterion (BIC) as a split criterion for an existing cluster as implemented in the R-based RPMM package.

#### Differentially methylated probes and differentially expressed genes

Differentially methylated probes and differentially expressed genes were obtained using TCGAbiolinks package in R [19]. Differentially methylated probes were obtained by comparing beta-values of probes between each methylation cluster and probes in normal samples. First, the mean methylation of each group for each probe was calculated, second, *p*-value was calculated using Wilcoxon test using Bonferroni adjustment method. The cutoff parameters were set to: absolute difference in methylation was larger than 0.2 and adjusted p-value less than 0.01. For obtaining differential gene expression general log-linearized model was used, with cutoff parameters: absolute fold change was larger than 1.0, and false discovery rate (FDR) adjusted p-value less than 0.01. For each cluster, we selected methylation probes mapping to promoter regions and had absolute methylation difference more than 0.3 compared to normal. We selected hypermethylated promoter probes (methylation difference more than 0.3) and down-regulated genes with logarithmic fold change of at least − 1.0. Our selection also included hypomethylated promoter probes (methylation difference was less than − 0.3) and up-regulated genes with logarithmic log fold change more than 1.0. This selection was overlapped among all resulting clusters to obtain the genes with aberrant methylation and differential expression common to all four clusters.

#### Data visualization, text mining and survival analysis

The HM450 DNA methylation β-values of 4165 most variable probes along with methylation cluster, location, gender, tumor stage, *MLH1* promoter methylation and mutations in *BRAF*, *KRAS*, *APC* and *TP53* were represented graphically using heatmap visualization from ComplexHeatmap package in R programming software [20]. For construction of protein-protein interaction networks the STRING database (version 10.5) was used which produces a functional association network, using interaction sources, such as text mining, experiments, database, co-expression, neighborhood, gene fusion and co/occurrence. To identify gene ontology processes enriched within our 198 set of genes from the intersection of all resulting clusters the STRING database was used [21]. We used the GeneRIF (Gene Reference into Function) database as the source text for finding gene-disease associations previously published and stored on PubMed system. We performed several queries using different conditions and terms such as: “cancer”, “colorectal”, “colon”, “methylation”, “expression” and identification numbers for all 198 genes. For the Cox proportional hazard model package survival in R software was used [22]. The influence of the different clinical and genetic parameters was determined with logrank test, where *p*-value was less than 0.05. Some hazard ratios could not be computed, since gene was up/down regulated or hypo/hypermethylated in all samples. Hazard ration can be computed when there are two groups.

### Experimental validation

#### Clinical samples

Samples used for experimental validation in our study are presented in Table 1. Our study was comprised of 115 samples, of which 90 were fresh frozen tissue samples and 25 tissue samples were stabilized in RNAlater solution (Ambion). All the samples (*n* = 115) were used in the methylation experiment, however, the latter 25 samples, that were stored in RNAlater, were of sufficient quality to be used also for gene expression experiment.

**Table 1:** Clinical data for samples used in validation

	Methylation set (*n* = 115) n (%)	Expression set (*n* = 25) n (%)
Gender		
Female	54 (47)	10 (40)
Male	61 (53)	15 (60)
Location		
Ascending colon	27 (23.5)	5 (20)
Transverse colon	14 (12.2)	3 (12)
Descending colon	6 (5.2)	1 (4)
Sigmoid colon	18 (15.7)	3 (12)
Rectum	50 (43.5)	13 (52)
T		
T1	12 (10.4)	1 (4)
T2	28 (24.3)	3 (12)
T3	61 (53.0)	20 (80)
T4	14 (12.2)	1 (4)
N		
N0	56 (48.7)	15 (60)
N1	53 (46.1)	5 (20)
N2	5 (4.3)	4 (16)
Nx	1 (0.9)	1 (4)
M		
M0	84 (73.0)	18 (72)
M1	13 (11.3)	2 (8)
Mx	18 (15.7)	5 (20)

*T* tumor size, *N* lymph node infiltration, *Nx* lymph node infiltration not determined, *M* distant metastasis, *Mx* distant metastasis not determined, *n* number of samples

The latter 25 samples were collected during surgical colectomy of patients diagnosed with primary colorectal adenocarcinoma. The patients’ whose samples were collected had no other cancer than CRC, and no previous radio- or chemotherapy. From each patient tumor and normal sample was collected, where normal samples of healthy colon mucosa were collected at least 20 cm away from tumor site. Both tumor and normal mucosa samples were placed in RNAlater solution, which stabilizes tissue and enables DNA and RNA extraction. Samples were submerged in RNAlater and incubated for 24 h at 4 °C to allow the solution to penetrate through the sample. After incubation period, the samples were stored at − 20 °C.

For all 115 samples data about gender, tumor location, size, nodal infiltration, distant metastasis, and survival data was obtained from Cancer Registry of Slovenia. Patients enrolled in the study signed an informed consent form agreeing to participate in the study. The National Medical Ethics Committee of the Republic of Slovenia approved this research.

#### RNA/DNA isolation

DNA and RNA from tissues stored in RNALater solution were isolated with All prep DNA/RNA Mini Kit (Qiagen), according to the manufacturer’s recommendations. DNA and RNA quantity and quality were determined spectrophotometrically by NanoDrop ND-1000 (Thermo Fisher Scientific). DNA (*n* = 90) was isolated from fresh frozen samples with QIAamp DNA Mini Kit (Qiagen), according to the manufacturer’s recommendations. DNA quantity and quality were determined spectrophotometrically by NanoDrop ND-1000 (Thermo Fisher Scientific).

#### Bisulfite conversion and MS-HRM experiment

After DNA extraction, 1000 ng of DNA was used in bisulfite conversion with innuCONVERT Bisulfite Basic Kit (Analytik Jena AG). Twenty ng of bisulfite converted DNA was used in methylation-sensitive high resolution melting experiment (MS-HRM). Primers for MS-HRM were designed in Methyl Primer Express Software v1.0 (Thermo Fisher Scientific) (Additional file 1: Table S1) to amplify both, methylated and unmethylated DNA. Amplicon length was designed to cover the specific CpG sites in the 5′ UTR region of selected genes differentially methylated from the bioinformatics analysis. For some genes one amplicon covers more than one CpG site. As controls, completely methylated and completely unmethylated commercially available bisulfite converted DNA (EpiTect PCR Control DNA Set, Qiagen) were used in each MS-HRM run, to help with assessment of methylation status of the samples. The amplification was performed using the following protocol: 2.00 μL bisulphite converted DNA, 1.00 μL of each primer, 0.50 μL dNTP, 1.00 μL HotStart Taq Plus Buffer (10x), 0.05 μL HotStart Taq Plus Polymerase (5 U/μL), and 0.3 μL Syto9 with Nuclease-free water to obtain a total PCR reaction volume of 10 μL. Optimized cycling protocol for HRM analysis on the Rotor-Gene Q (Qiagen) was preformed including: initial denaturation at 95 °C for 5 min; 45 times at 94 °C for 15 s, annealing temperature (Additional file 1: Table S1) for 30 s, extension at 72 °C for 30 s (using Fluorescence data acquisition on the “HRM” channel at this step). HRM analysis was performed immediately after PCR under the following conditions: 60–99 °C with 0.1 °C ramp rate. This step requires fluorescence data acquisition on the “HRM” channel. All amplifications were performed in duplicate, using Rotor-Gene Q (Qiagen), following the manufacturer’s recommendations.

#### Reverse transcription and qPCR experiment

Gene expression levels were determined using SYBR Green-based quantitative polymerase chain reaction (qPCR), which was performed on Rotor-Gene Q (Qiagen) detection system. All the reagents were from Qiagen, except where otherwise indicated. For investigated genes and four endogenous controls (*ACTB*, *GAPDH*, *RNN18S*, and *RPL13A*) in qPCR experiment, primers were all predesigned and used according to manufacturer’s instructions (Qiagen) (Additional file 2: Table S2).

Total RNA (300 ng) was reverse transcribed using QuantiTect Reverse Transcription Kit according to manufacturer’s instructions (Qiagen). The resulting cDNA was diluted 100-fold, and 3 μl was used for each qPCR reaction in 10 μl PCR master mix (5 μl 2x QuantiTect SYBR Green PCR Master Mix, 1 μl of forward and 1 μl of reverse primer). All the qPCR reactions were performed in duplicates or triplicates. The signal was collected at the endpoint of every cycle. Following amplification, melting curves analysis of PCR products were acquired on the SYBR channel using a ramping rate of 1 °C/60 s for 60–95 °C.

For expression calculation, geometrical average of threshold cycle (C_t_) of four endogenous controls (*ACTB*, *GAPDH*, *RNN18S*, and *RPL13A*) was subtracted from ct of investigated gene to obtain the difference of threshold cycles ΔC_t_. The comparative threshold cycles (ΔΔC_t_) were obtained by subtracting ΔC_t_ of tumor sample from ΔC_t_ of paired normal sample. The comparative threshold cycle is comparable with logFC, which is used for easier comparison with bioinformatics data.

## Results

### Study design

**Fig. 1:**
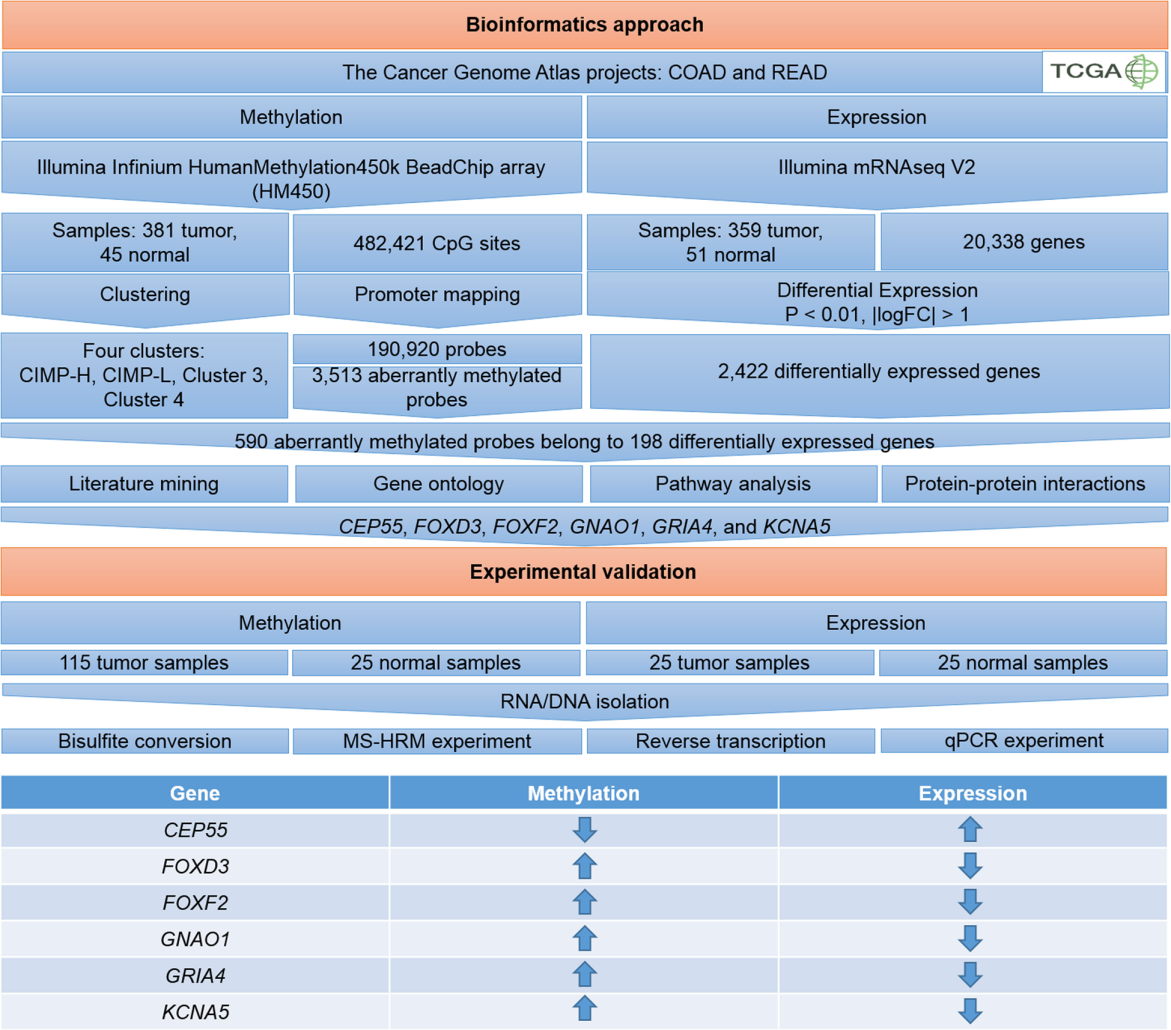
Workflow of the study. Bioinformatics approach was composed of DNA methylation and gene expression analysis. DNA methylation data was obtained from experiment using Illumina Infinium HumanMethylation450k BeadChip (HM450) array from 381 tumor samples and 45 normal samples. Unsupervised clustering of tumor samples resulted in four clusters (CIMP-H, CIMP-L, Cluster 3 and Cluster 4). Probes of each cluster were compared to probes in normal samples group with Wilcoxon test to obtain differentially methylated probes. HM450 array contains 482,421 CpG sites, of which 190,920 CpG probes are located in promoter regions. The intersect of differentially methylated probes among all four methylation clusters and probes located in promoter region resulted in 3513 probes. Gene expression data was obtained from Illumina mRNAseq V2 experiment, which contains gene expression for 20,338 genes. From 381 tumor samples used in methylation analysis, 359 had gene expression data. For comparison of gene expression, 51 normal samples were used. Using gene expression data, tumor samples were divided into the same clusters as samples of methylation data and each cluster was compared to normal group using general log-linearized model to obtain differentially expressed genes. Intersect among all clusters in expression analysis gave 2422 differentially expressed genes. The bioinformatics approach resulted in 590 differentially methylated probes belonging to 198 differentially expressed genes, which exhibit hypermethylation/down-regulation or hypomethylation/up regulation. After literature mining, gene ontology, pathway analysis and protein-protein interactions we selected *CEP55*, *FOXD3*, *FOXF2*, *GNAO1*, *GRIA4* and *KCNA5* for further experimental validation. For experimental validation we used 115 samples in methylation experiment and 25 samples in gene expression experiment. After RNA/DNA isolation, DNA was bisulfite converted and used in methylation-sensitive high-resolution melt experiment (MS-HRM), and RNA was reverse transcribed to cDNA and used in quantitative real-time PCR (qPCR) experiment. Gene *CEP55* was hypomethylated and up regulated, while *FOXD3*, *FOXF2*, *GNAO1*, *GRIA4* and *KCNA5* were hypermethylated and down-regulated, consistent with our bioinformatics analysis. Figure was prepared in Microsoft Power Point software. Legend: COAD, Colon adenocarcinoma; READ, rectum adenocarcinoma; logFC, logarithm of fold change

The study consisted of two major parts – bioinformatics analysis and experimental validation (Fig. 1). Bioinformatics analysis was performed on samples from projects COAD and READ obtained from TCGA. Methylation data was collected by experiment with HM450, which is the most comprehensive methylation data collection available on TCGA. Methylation data were obtained on the HM450 platform of 381 tumor tissue samples, together with 45 normal samples. HM450 covers 482,421 CpG sites within the genome, which were mapped to regulatory regions that are likely to be involved in gene regulation: the open chromatin region, predicted enhancer region, predicted promoter, predicted promoter flanking region and transcription factor binding site. Altogether we obtained 190,920 probes located in 81,467 regulatory regions. Specifically, 14,718 probes mapped into the open chromatin region, 9513 into the enhancer, 122,576 into the promoter, 42,150 into the promoter flanking region, and 13,670 probes mapped into the transcription factor binding site. Some regulatory regions can overlap, so some probes belong to more than one regulatory region.

Unsupervised clustering on methylation data resulted in four clusters. Probes in samples of each cluster were compared to probes of normal samples to obtain significant differentially methylated probes for each cluster. Differentially methylated probes with significant *p*-values of each cluster were intersected among all clusters. The location of the intersected probes was compared to a list of 190,920 probes in regulatory regions, which resulted in 3513 probes that were both in intersect among clusters and in regulatory regions. Gene expression data of the same samples were available from an Illumina mRNAseq V2 experiment. From the set of 381 tumor samples used in methylation analysis, gene expression data were available for 359 tumor samples. The samples in gene expression of each cluster was then compared to gene expression of 51 normal samples. The intersect among all four clusters gave 2422 differentially expressed genes. Integrating methylation in regulatory regions and gene expression data, considering hypermethylation/down-regulation and hypomethylation/up regulation combinations, resulted in 590 aberrantly methylated probes belonging to 198 genes. The resulting 590 aberrantly methylated probes belong to 373 regulatory regions, in which 72 probes are located in the open chromatin region, 5 probes in the enhancer, 439 probes in the promoter, 97 probes in the promoter flanking region and 66 probes in the transcription factor binding site.

The second part of the study consisted of experimental validation of six selected genes. For this purpose, we tested DNA methylation status on 115 tumor tissue samples, of which 25 were paired tumor and normal tissue samples of sufficient quality for both DNA methylation and gene expression experiment.

### Clustering of methylation data

**Fig. 2:**
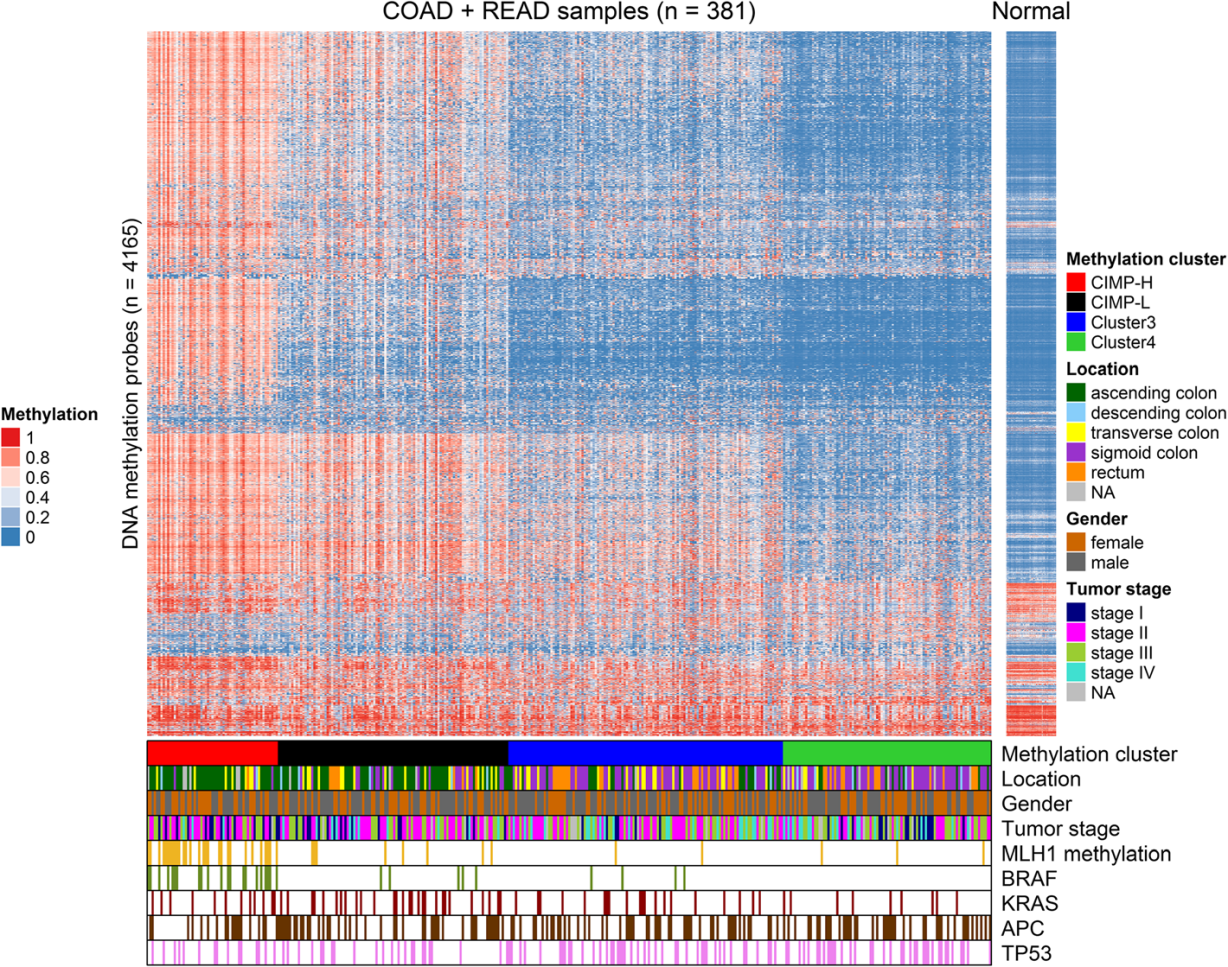
Heatmap representing methylation, some clinical and genetic data. RPMM-based classification of 381 of tumor samples from colon adenocarcinoma (COAD) and rectum adenocarcinoma (READ) project in TCGA and heatmap representation of HM450 DNA methylation data. DNA methylation profiles of 4165 probes with most variable DNA methylation values (standard deviation > 0.25) in the 381 colorectal tumor sample set. The DNA methylation β-values are represented by a color scale from blue (low DNA methylation) to red (high DNA methylation). The probes are arranged based on the order of unsupervised hierarchal cluster analysis. Four subgroups were derived by RPMM-based clustering and are indicated below the heatmap: (red) CIMP-H cluster (*n* = 59); (black) CIMP-L cluster (*n* = 104); (blue) Cluster 3 (*n* = 124); (green) Cluster 4 (*n* = 94). Below methylation cluster is location: (dark green) ascending colon, (light blue) descending colon, (yellow) transverse colon, (purple) sigmoid colon, (orange) rectum, (grey) NA – data not available; gender: (light brown) female, (dark gray) male; tumor stage: (navy blue) stage I, (pink) stage II, (light green) stage III, (turquoise) stage IV, (gray) NA – data not available; *MLH1* methylation (gold bars), *BRAF* mutation (olive bars), *KRAS* mutation (dark red bars), *APC* mutation (dark brown bars) and *TP53* mutation (violet bars). On the right side of the heatmap are methylation levels of the samples from normal colorectal mucosa (*n* = 45) for comparison. The average age of patients is 64.2 and the average age of patients with normal mucosa is 68.8. Figure was prepared using library ComplexHeatmap in R programming software

Unsupervised clustering analysis was performed on the methylation data of 381 samples from COAD and READ. The clustering resulted in four separate clusters, denoted CIMP-H, CIMP-L, Cluster 3 and Cluster 4, according to the names used in the literature [10] (Fig. 2). As established previously, CIMP-H CRCs have a higher rate of hypermethylated promoter of the *MLH1* gene and higher mutation rate in gene *BRAF*. Similarly, we found *MLH1* hypermethylation is present in 49.2% of samples in CIMP-H but only 13 samples out of 222 (5.8%) from the other three clusters combined (Table 1). Almost all mutations in gene *BRAF* were found in the CIMP-H cluster (35.6%), a few were found in CIMP-L (4.8%) and Cluster 3 (3.2%), while none were found in Cluster 4. Cluster CIMP-L was characterized by a high frequency of *KRAS* mutations, with rare mutations in *BRAF*, and low rate of *TP53* mutations. Indeed, the rate of *KRAS* mutations in this cluster was the highest (26.9%) although not nearly as high as reported in the literature (92%). Mutations in *TP53* were found in 22.1% of samples (Table 2). CIMP-H and CIMP-L clusters are both associated with tumor presence in ascending colon, where in our case, tumor presence in the ascending colon was 73.6% in CIMP-H and 52.5% in CIMP-L.

**Table 2:** Genetic and clinical features of all samples from TCGA and samples from TCGA belonging to each of the four methylation-based clusters

	CIMP-H (*n* = 59) n (%)	CIMP-L (*n* = 104) n (%)	Cluster 3 (*n* = 124) n (%)	Cluster 4 (*n* = 94) n (%)	ALL (*n* = 381) n (%)
APC/WT	19/40 (32.2/67.8)	43/61 (41.3/58.7)	49/75 (39.5/60.5)	42/52 (44.7/55.3)	153/228 (40.2/59.8)
KRAS/WT	12/47 (20.3/79.7)	28/76 (26.9/73.1)	20/104 (16.1/83.9)	9/85 (9.6/90.4)	69/312 (18.1/81.9)
TP53/WT	15/44 (25.4/74.6)	23/81 (22.1/77.9)	43/81 (34.7/65.3)	37/57 (39.4/60.6)	118/263 (31.0/69.0)
BRAF/WT	21/38 (35.6/64.4)	5/99 (4.8/95.2)	4/120 (3.2/96.8)	0/94 (0.0/100.0)	30/351 (7.9/92.1)
MLH1 Methylated/Unmethylated	29/30 (49.2/50.8)	8/96 (7.7/92.3)	2/122 (1.6/98.4)	3/91 (3.2/96.8)	42/339 (11.0/89.0)
Gender Female/Male	30/29 (50.8/49.2)	46/58 (44.2/55.8)	51/73 (41.1/58.9)	47/47 (50.0/50.0)	174/207 (45.7/54.3)
Location					
Ascending colon	39 (73.6)	53 (52.5)	23 (18.7)	10 (11.0)	125 (34.2)
Transverse colon	8 (15.1)	16 (15.8)	18 (14.6)	6 (6.6)	48 (13.2)
Descending colon	2 (3.8)	5 (5.0)	2 (1.6)	5 (5.5)	14 (3.8)
Sigmoid colon	3 (5.7)	13 (12.9)	34 (27.6)	36 (39.6)	86 (23.6)
Rectum	1 (1.9)	14 (13.9)	46 (37.4)	31 (34.1)	92 (25.2)
No data	6	3	1	3	16
T					
T1	2 (3.4)	2 (1.9)	4 (3.3)	3 (3.2)	11 (2.9)
T2	12 (20.3)	18 (17.3)	7 (5.7)	15 (16.1)	52 (13.7)
T3	37 (62.7)	71 (68.3)	91 (74.0)	67 (72.0)	266 (70.2)
T4	7 (11.9)	13 (12.5)	21 (17.1)	8 (8.6)	49 (12.9)
Tis	1 (1.7)				1 (0.3)
No data			1	1	2
N					
N0	38 (64.4)	63 (60.6)	60 (48.8)	43 (46.2)	204 (53.8)
N1	12 (20.3)	22 (21.2)	40 (32.5)	29 (31.2)	103 (27.2)
N2	9 (15.3)	19 (18.3)	21 (17.1)	21 (22.6)	70 (18.5)
Nx			2 (1.6)		2 (0.5)
No data			1	1	2
M					
M0	46 (79.3)	71 (69.6)	83 (68.0)	58 (63.7)	258 (69.2)
M1	3 (5.2)	11 (10.8)	23 (18.9)	16 (17.6)	53 (14.2)
Mx	9 (15.5)	20 (19.2)	16 (13.1)	17 (18.7)	62 (16.6)
No data	1	2	2	3	8
MSI					
MSI-H	28 (47.5)	12 (11.5)	6 (4.8)	7 (7.5)	53 (14.0)
MSI-L	9 (15.3)	23 (22.1)	16 (12.9)	14 (15.1)	62 (16.4)
MSS	22 (37.3)	68 (65.4)	102 (82.3)	72 (77.4)	264 (69.7)
Indeterminate		1		1	2
Stage					
Stage I	14 (24.1)	17 (17.0)	6 (5.2)	15 (17.0)	52 (14.5)
Stage II	24 (41.4)	43 (43.0)	48 (41.4)	23 (26.1)	138 (38.1)
Stage III	17 (29.3)	29 (29.0)	40 (34.5)	32 (36.4)	118 (32.6)
Stage IV	3 (5.2)	11 (11.0)	22 (19.0)	18 (20.5)	54 (14.9)
No data	1	4	8	6	19

*CIMP* the CpG island methylator phenotype, *n* number of samples, *MUT* mutation, *WT* wild-type, *MSI* microsatellite instability, *MSS* microsatellite stable, *T* tumor size, *N* lymph node infiltration, *Nx* lymph node infiltration not determined, *M* distant metastasis, *Mx* distant metastasis not determined

The both non-CIMP clusters, Cluster 3 and Cluster 4, had lower frequencies in mutations in *BRAF* (3.2 and 0%) and *KRAS* (16.1 and 9.6%), respectively. There was a higher rate of *TP53* mutations, 34.7% in Cluster 3 and 39.4% in Cluster 4. Both of these clusters had a higher rate of tumor presence in sigmoid colon, 27.6% of samples in Cluster 3 and 39.6% in Cluster 4, and rectum, 37.4% in Cluster 3 and 34.1% in Cluster 4. High microsatellite instability was more pronounced in CIMP-H (47.5%), while low microsatellite instability was most frequent in CIMP-L (22.1%). Microsatellite stability was predominant in Cluster 3 (82.3%) and Cluster 4 (77.4%) but it is also quite high in CIMP-L cluster (65.4%). Fig. 2 shows there is no distinct feature (i.e., mutation, promoter methylation) common to all samples in any cluster or common to all clusters.

### Aberrantly methylated probes and differentially expressed genes

Our analysis resulted in 590 aberrantly methylated probes found at the intersect between clusters and mapped to regulatory regions. These probes belong to 198 differentially expressed genes, which were differentially expressed in each cluster when compared to normal tissue samples (Additional file 3: Table S3). Using these 198 protein-coding genes, we performed protein-protein interaction network (PPIN), functional and literature mining analysis.

The 198 differentially expressed genes were uploaded to the STRING database to construct a PPIN (Additional file 4: Figure S1). Since genes were selected based on aberrant methylation and differential gene expression present in all four clusters, some of the proteins coded by those genes are connected to networks, while others do not interact. To get some information about the biological functions of selected genes, we conducted gene ontology and pathway analysis (Additional file 5: Table S4). Pathway analysis reviled 12 significant KEGG pathways, of which the first three were neuroactive ligand-receptor interaction, cholinergic synapse and circadian entrainment. The circadian entrainment pathway has previously been associated with rectum adenocarcinoma [23]. We therefore selected two genes, *GRIA4* and *GNAO1*, involved in this pathway, as well as gene *KCNA5*. KCNA5 is indirectly associated with this pathway through neighboring proteins KCNIP1 to NOS1. According to our PPIN analysis, there is another set of proteins with many interactions, of which BMP4 is the hub, which is involved in Hedgehog and TGF-beta signaling pathways. Moreover, our literature mining analysis revealed that the expression of gene *BMP4* had already been studied (Additional file 6: Table S5), so we decided to select the genes *FOXD3* and *FOXF2* for experimental validation, whose proteins interact with BMP4 and both of which are transcription factors. We selected gene *CEP55* on the basis of hypomethylation/up regulation, which is involved in biological process of cell cycle.

**Fig. 3:**
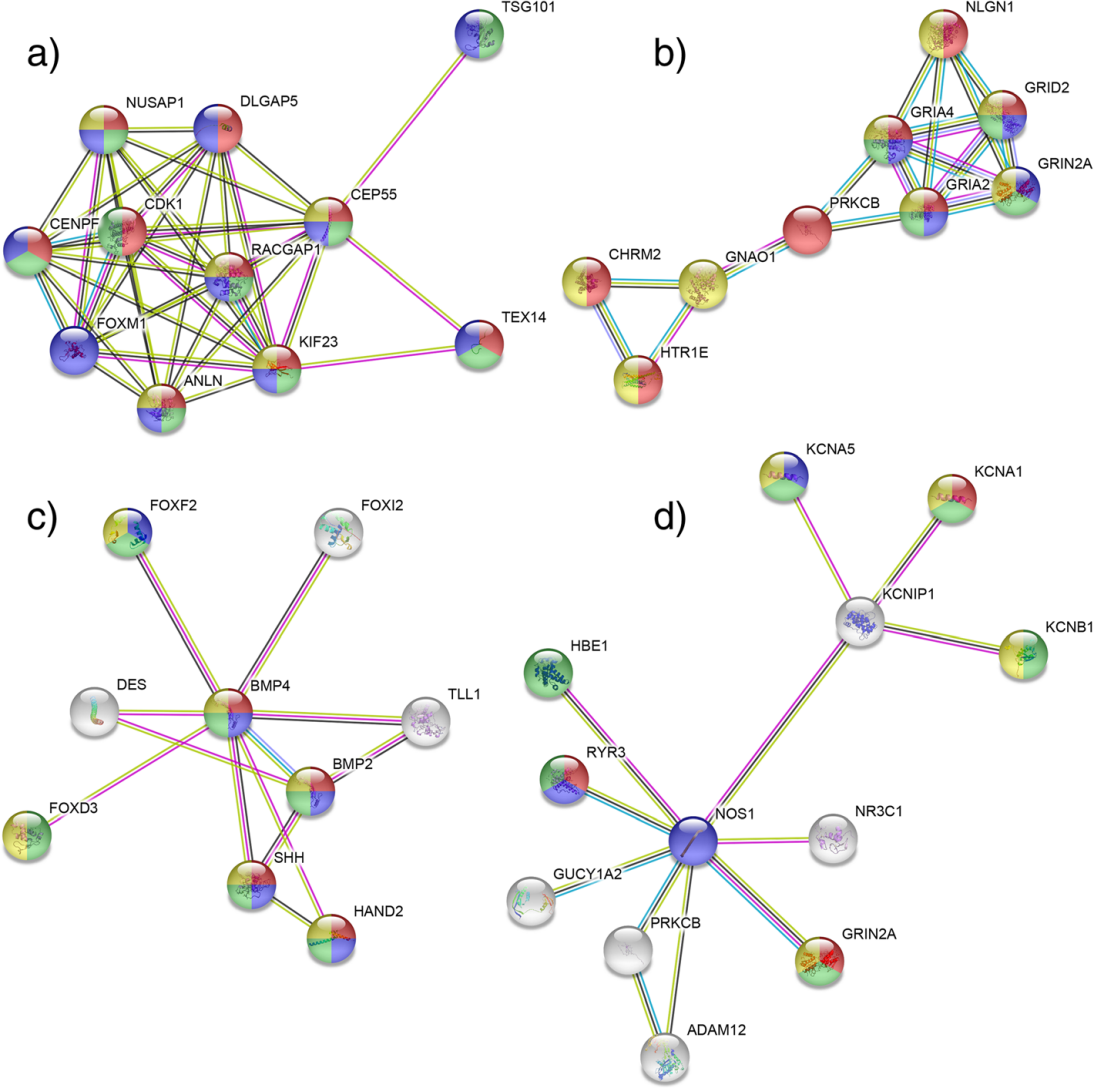
Protein-protein interaction networks and presentation of biological processes from Gene ontology. Lines: (cyan) from curated databases, (magenta) experimentally determined, (green) gene neighborhood, (red) gene fusions, (blue) gene co-occurrence, (yellow green) texmining, (black) co-expression, (purple) protein homology; circles: proteins; **a**) protein-protein network for *CEP55* gene: (red) GO:0007067 mitotic nuclear division, (blue) GO:0051301 cell division, (green) GO:0022402 cell cycle process, (yellow) GO:0000281 mitotic cytokinesis; **b**) protein-protein network for *GNAO1* and *GRIA4* gene: (red) GO:0007268 synaptic transmission, (blue) GO:0035235 ionotropic glutamate receptor signaling pathway, (green) GO:0035249 synaptic transmission, glutamatergic, (yellow) GO:0007165 signal transduction; **c**) protein-protein network for *FOXD3* and *FOXF2* gene: (red) GO:0003156 regulation of organ formation, (blue) GO:0048762 mesenchymal cell differentiation, (green) GO:0048863 stem cell differentiation, (yellow) GO:0048568 embryonic organ development; **d**) protein-protein network for *KCNA5* gene: (red) GO:0071286 cellular response to magnesium ion, (blue) GO:0051481 negative regulation of cytosolic calcium ion concentration, (green) GO:0051259 protein oligomerization, glutamatergic, (yellow) GO:0001508 action potential. Figure was downloaded from STRING web application (string-db.org)

We constructed child PPIN with the six selected proteins described above, presented in Fig. 3, whereby b), c) and d) were constructed using the neighboring proteins that are coded by genes from our list, and a) was constructed with the first interacted protein added, since CEP55 had no interactions in PPIN constructed from our gene set. Gene *CEP55* is involved in cell division and the cell cycle process, *GNAO1* and *GRIA4* participate in signal transduction, *FOXD3* and *FOXF2* take part in stem cell differentiation and embryonic organ development, while *KCNA5* is involved in negative regulation of cytosolic calcium ion concentration, protein oligomerization and action potential.

According to our in silico analysis, gene *CEP55* was hypomethylated and up-regulated, while the other five genes, *FOXD3*, *FOXF2*, *GNAO1*, *GRIA4* and *KCNA5*, were hypermethylated and down-regulated (Table 3). The genes expressed various levels of difference in methylation and expressions. The most down-regulated gene, regardless of cluster, was *FOXD3* (logFC = − 3.05). It was down-regulated in 98.3% of samples. The methylation difference was high (0.4), although present in fewer samples (91.9%). The highest methylation difference was present in all four promoter probes of gene *GRIA4* (from 0.46 to 0.54), with two of them present in 98.4% of samples. Regardless of cluster, *GRIA4* was down-regulated (logFC = − 2.41) in 98.1% of samples.

**Table 3:** Differentially methylated probes and differentially expressed genes from TCGA (*p*-values < 0.01), Δβ represents difference in methylation and logFC differential gene expression

Gene	CpG	CIMP-H		CIMP-L		Cluster 3		Cluster 4		All		All	All
		Δβ	logFC	Δβ	logFC	Δβ	logFC	Δβ	logFC	Δβ	logFC	Changed Methylation (*n* = 381)	Changed Expression (*n* = 359)
*CEP55*	cg25314624	−0.36	1.62	−0.38	1.38	−0.38	1.24	−0.35	1.18	0.37	1.32	376 (98.7%)	341 up (95.0%)
*FOXD3*	cg15617155	0.31	−4.28	0.31	−3.21	0.33	−2.70	0.32	−2.53	0.40	− 3.05	373 (97.9%)	353 down (98.3%)
*FOXF2*	cg12221475	0.34	−2.55	0.41	−2.09	0.39	−1.85	0.41	−1.58	0.39	−1.96	309 (81.1%)	355 down (98.9%)
*GNAO1*	cg00866976	0.35	−4.04	0.39	−3.18	0.37	−2.69	0.41	− 2.04	0.38	− 2.88	304 (79.8%)	352 down (98.1%)
	cg10273340	0.40		0.44		0.42		0.46		0.43		306 (80.3%)	
*GRIA4*	cg00343633	0.52	−3.12	0.55	−2.59	0.55	−2.19	0.51	−2.03	0.54	−2.41	375 (98.4%)	352 down (98.1%)
	cg03225817	0.44		0.49		0.49		0.45		0.47		367 (96.3%)	
	cg07972135	0.44		0.50		0.51		0.47		0.49		369 (96.9%)	
	cg23559689	0.43		0.48		0.47		0.46		0.46		375 (98.4%)	
*KCNA5*	cg16897114	0.34	−2.81	0.35	−2.24	0.35	−1.97	0.34	−1.32	0.35	−2.02	358 (94.0%)	354 down (98.6%)

*CIMP* the CpG island methylator phenotype, *logFC* logarithm of fold change, *Δβ* difference in average beta value between tumor and normal mucosa samples, *n* number of samples

We evaluated the aberrant methylation of regulatory region per sample. In cases of *GNAO1* and *GRIA4*, with which there was more than one methylation probe per gene, at least one of the probes had to be hypermethylated to conclude that the section was hypermethylated. No sample had less than two markers aberrantly methylated. There were two samples (0.5%) with two markers and six samples (1.6%) with three markers aberrantly methylated, 97.9% samples had at least four markers aberrantly methylated. Specifically, 33 samples (8.7%) had four, 88 samples (23.1%) had five and 252 samples (66.1%) had all six markers aberrantly methylated.

To test the clinical data available in TCGA, we performed survival analysis using the Cox proportional hazards model (Table 4). Using univariate analysis, we found that late stage compared to early stage tumor has the highest hazard ratio. The second highest hazard ratio was observed with the presence metastasis, followed by presence of polyps and age above 60. By multivariate analysis, we obtained two significant hazard ratios, presence of metastasis and age above 60. Overall model was significant, with *p*-value 2.475e-06.

**Table 4:** Cox proportional hazards model on TCGA dataset

	Univariate analysis			Multivariate analysis		
	hazard ratio	95% confidence interval	*p*-value	hazard ratio	95% confidence interval	*p*-value (*p*-value of model)
Cluster (CIMP vs. Non-CIMP (*n* = 180)	1.182	0.6763–2.065	0.5593			
Age (≥60) (*n* = 180)	2.328	1.193–4.544	0.007842	3.693 (*n* = 155)	1.634–8.349	0.00169 (2.475e-06)
Polyps present (*n* = 138)	2.518	1.03–6.155	0.03877			
Gender (Male vs. Female) (*n* = 180)	1.662	0.930–2.967	0.07991			
Location (rectum vs. Colon) (*n* = 176)	1.07	0.4935–2.318	0.8656			
Stage (III/IV vs. I/II) (*n* = 176)	1.95	1.073–3.543	0.0251			
Metastasis presence (*n* = 155)	3.398	1.82–6.343	0.0002934	3.813 (*n* = 155)	2.032–7.156	3.08e-05 (2.475e-06)
Lymph node infiltration (*n* = 179)	1.725	0.978–3.042	0.05665			
Tumor size (T3/T4 vs. T1/T2) (*n* = 179)	1.746	0.6899–4.417	0.206			
MSI vs. MSS (*n* = 179)	1.076	0.5948–1.947	0.8092			
Late stage vs. early stage (*n* = 164)	3.876	0.9389–16	0.04349			
Methylation (methylated vs unmethylated) (*n* = 165)						
*CEP55* - cg25314624	1.3444	0.3213–5.626	0.6842			
*FOXD3* – cg15617155	ND					
*FOXF2* - cg12221475	1.893	0.8455–4.236	0.1146			
*GNAO1*- cg00866976	0.8484	0.4304–1.673	0.6347			
cg10273340	0.8404	0.4161–1.697	0.6275			
*GRIA4* - cg00343633	0.4816	0.1483–1.563	0.2138			
cg03225817	0.6222	0.1924–2.012	0.4238			
cg07972135	0.6185	0.2206–1.734	0.3564			
cg23559689	0.6887	0.1662–2.853	0.605			
*KCNA5* - cg16897114	1.91	0.4558–8.002	0.3682			
Expression (upregulated vs down-regulated)						
CEP55	1.089	0.3344–3.549	0.8871			
FOXD3	1.859	0.4483–7.711	0.3853			
FOXF2	ND					
GNAO1	0.9207	0.2222–3.815	0.9092			
GRIA4	3.409	0.8084–14.38	0.07561			
KCNA5	1.859	0.4483–7.711	0.3853			

*CIMP* the CpG island methylator phenotype, *n* number of samples, *MSI* microsatellite instability, *MSS* microsatellite stable, *ND* not determinable

### Experimental validation

Based on the bioinformatics analysis results described above, we experimentally validated six selected genes. Experimental validation of the methylation results of the in silico analysis was performed using a larger cohort of samples (n = 115). Results revealed *CEP55* to be hypomethylated in 97.3% of CRC cases and *FOXD3*, *FOXF2*, *GNAO1*, *GRIA4* and *KCNA5* being hypermethylated in 100, 90.2, 100, 97.3 and 99.1% of CRC cases, respectively.

**Fig. 4:**
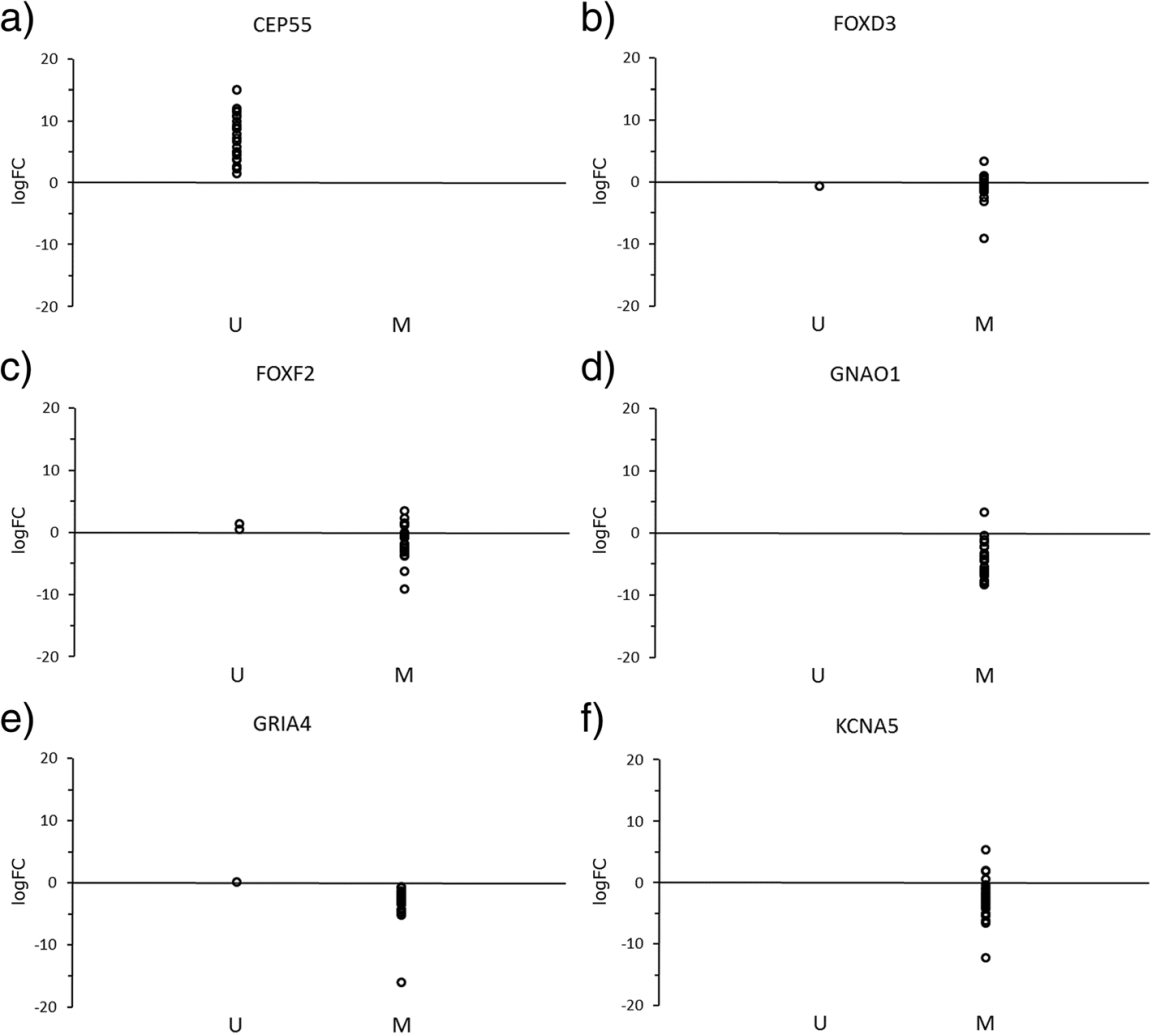
Gene expression and methylation status of *CEP55, FOXD3, FOXF2, GNAO1, GRIA4*, and *KCNA5* in our samples. For the 25 samples used in methylation and gene expression experiment, results are shown for gene: **a**) *CEP55*, **b**) *FOXD3*, **c**) *FOXF2*, **d**) *GNAO1*, **e**) *GRIA4*, and **f**) *KCNA5*. Figure was prepared in Microsoft Excel software. Legend: logFC, logarithm of fold change; U, unmethylated; M, methylated

The methylation and expression profile of 25 samples on which both experiments could be performed are shown on Fig. 4. Consistent with the bioinformatics analysis, our experimental data on expression analysis on 25 RNAlater stored samples showed an overall expression of gene *CEP55* as up-regulated (logFC = 7.47, *p* <  0.001), while *FOXD3* (logFC = − 0.66, *p* = 0.027), *FOXF2* (logFC = − 1.33, *p* = 0.021), *GNAO1* (logFC = − 4.78, *p* <  0.001), *GRIA4* (logFC = − 3.25, *p* <  0.001) and *KCNA5* (logFC = − 2.81, *p* <  0.001) were down-regulated in CRC compared to corresponding normal mucosa.

Methylation analysis on the same cohort of samples (*n* = 25) revealed that, in CRC compared to normal mucosa, gene *CEP55* was completely hypomethylated and up-regulated. Gene *GRIA4* had one sample hypomethylated and slightly up-regulated, all other samples were hypermethylated and down-regulated. Gene *GNAO1* had one sample that was hypermethylated and up-regulated, all the other samples were hypermethylated and down-regulated. We obtained mixed results for *FOXD3*, *FOXF2*, and *KCNA5* genes. *FOXD3* had one sample hypomethylated and down-regulated, while seven samples were hypermethylated and up-regulated, the rest being hypermethylated and down-regulated. *FOXF2* had two samples hypomethylated and up-regulated, four hypermethylated and up-regulated, while the others were hypermethylated and down-regulated. *KCNA5* had no hypomethylated samples; four samples were hypermethylated and up-regulated, while the rest were hypermethylated and down-regulated.

Comparing the expression and methylation status of RNAlater stored samples (*n* = 25) showed that gene *GNAO1* had the most down-regulated gene expression and was also hypermethylated in all samples (Table 5). Gene *GRIA4* showed hypermethylation and down-regulation in 97.3 and 95.2% of samples, respectively. Up-regulation in all samples and hypomethylation in 97.3% of samples was observed in gene *CEP55*. The performance of other genes in terms of the correlation of their expression to methylation status was less encouraging, with *FOXD3*, *FOXF2,* and *KCNA5* exhibiting down-regulation in fewer than 90% of samples, even only 68% for *FOXF3*.

**Table 5:** Methylation and gene expression on our dataset. *P*-value was calculated between ΔC_t_ of tumor and ΔC_t_ of normal mucosa samples

Gene	Expression dataset (*n* = 25)			Methylation dataset (*n* = 115)
	Methylation	Expression	logFC (*p*-value)	Methylation
*CEP55*	100% Unmethylated	100% up-regulated	7.47 (< 0.001)	97.3% unmethylated
*FOXD3*	96% methylated	68% down-regulated	−0.66 (0.027)	100% methylated
*FOXF2*	88% methylated	76% down-regulated	−1.33 (0.021)	90.2% methylated
*GNAO1*	100% methylated	96% down-regulated	−4.78, (< 0.001)	100% methylated
*GRIA4*	96% methylated	95.2% down-regulated	−3.25 (< 0.001)	97.3% methylated
*KCNA5*	100% methylated	84% down-regulated	−2.81 (< 0.001)	99.1% methylated

*n* number of samples, *logFC* logarithm of fold change

Additionally, the number of aberrant methylation markers of six genes *CEP55*, *FOXD3*, *FOXF2*, *GNAO1*, *GRIA4* and *KCNA5* per sample was noted. There were no samples with fewer than three markers aberrantly methylated. Three samples (2.6%) had three markers aberrantly methylated. There were 97.4% of samples with at least four markers aberrantly methylated. Specifically, 11 samples (9.6%) had four, 22 samples (19.1%) had five and 79 samples (68.7%) had all six markers aberrantly methylated.

To test our clinical data, we performed survival analysis using the Cox proportional hazards model (Table 6). Using univariate analysis, we found that the presence of metastasis had the highest hazard ratio, followed by cancer progression, both with the highest significance. The next two highest hazard ratios were tumor size and lymph node infiltration, with lower significance than the previous two. By multivariate analysis, we obtained four significant hazard ratios, presence of metastasis, age above 60, lymph node infiltration and cancer progression. The overall model had a *p*-value significance of 5.453e-04.

**Table 6:** Cox proportional hazards model for our dataset. In each calculation number of samples is 115

*n* = 115	Univariate analysis			Multivariate analysis		
Clinical data	Hazard ratio	95% confidence interval	*p*-value	Hazard ratio	95% confidence interval	*p*-value (*p*-value of model)
Age (≥60)	1.967	1.01–3.833	0.043	2.825	1.330–6.000	0.007 (8.737e-12)
Gender (Male vs. Female)	1.544	0.982–2.426	0.058			
Metastasis presence	6.900	3.392–14.04	8.982e-10	6.692	3.137–14.278	8.8e-07 (8.737e-12)
Lymph node infiltration	2.016	1.367–2.975	3.366e-4	2.234	1.352–3.691	0.002 (8.737e-12)
Tumor size (T3/T4 vs. T1/T2)	1.867	1.143–3.048	0.011			
Location (rectum vs. colon)	1.797	1.139–2.836	0.011	2.047	1.212–3.457	0.007 (8.737e-12)
Late stage vs. early stage	1.926	1.172–3.165	0.009			
RT	1.134	0.534–2.409	0.743			
Chemotherapy	1.148	0.653–2.018	0.632			
Cancer progression	2.573	1.583–4.184	8.385e-05			
Methylation (methylated vs unmethylated)						
CEP55	1.276	0.312–5.217	0.734			
FOXD3	ND					
FOXF2	1.335	0.579–3.077	0.500			
GNAO1	ND					
GRIA4	0.339	0.106–1.092	0.05729			
KCNA5	0.0704	0.009–0.572	0.001			
Expression (upregulated vs down-regulated)						
CEP55	ND					
FOXD3	1.664	0.485–5.702	0.413			
FOXF2	0.267	0.034–2.086	0.176			
GNAO1	4.277	0.4992–36.65	0.148			
GRIA4	4.478	0.4999–40.11	0.142			
KCNA5	3.699	0.9577–14.29	0.042			

*ND* not determinable

## Discussion

The vast database of experimental data (TCGA) was used in our bioinformatics study. Interestingly, we observed that some samples have no mutations in the most commonly mutated tumor suppressors and oncogenes *BRAF*, *KRAS*, *TP53*, *APC*, nor do they exhibit methylation in promoter of the *MLH1* gene. According to the data from the literature, only 30% of CRCs harbor the *KRAS* mutation, 8–15% of CRCs the *BRAF* mutation, 60% of CRCs the *APC* mutation, up to 40–50% of CRCs the *TP53* mutation and 10–15% CRCs *MLH1* promoter methylation. Furthermore, we observed that neither *BRAF* mutations nor methylation in the *MLH1* promoter can accurately describe the CIMP-H cluster, since a change in *BRAF* was present in roughly one third and *MLH1* methylation in roughly 50% of samples in CIMP-H. Both changes also had a small presence also in the other three clusters.

In search of a common epigenetic change, we performed bioinformatics study of methylation and expression analysis based on TCGA data, which resulted in 198 sets of genes. We performed pathway and gene ontology analysis on these genes. Pathway analysis resulted in 12 significant KEGG pathways, of which the first four were neuroactive ligand-receptor interaction, cholinergic synapse, circadian entrainment and calcium signaling pathway. These pathways included altogether 26 of our genes. Gene Ontology resulted in 137 significant biological processes, in which all the pathways together included 155 genes from our list. The first most significant biological process pathways were mostly related to the nervous system and its development in one part, and in the other part pathways were related to cell differentiation, adhesion and development. Gene ontology molecular function revealed that genes in our selected set are involved in receptor activity, whereas the gene ontology cellular component showed that most of genes in our set are part of plasma membrane.

Among prominent identified genes were *CEP55*, involved in the cell cycle process, *FOXD3* and *FOXF2*, which are involved in stem cell differentiation, *GNAO1* and *GRIA4*, which participate in signal transduction and *KCNA5*, which is a part of the regulation of the calcium ion concentration*.* We experimentally validated these six genes on our own CRC tissue samples, confirming the prediction of expression and methylation status. Using both approaches, we found gene *CEP55* to be hypomethylated and up-regulated, while the other five genes, *FOXD3*, *FOXF2*, *GNAO1*, *GRIA4* and *KCNA5*, were hypermethylated and down-regulated. A number of studies have already described *CEP55* as an overexpressed gene in cancer tissue samples. It maps to chromosomal regions 10q23 and encodes centrosome- and midbody-associated protein [24]. It is the latest member discovered in the centrosomal relative protein family and it has an important role in cell mitosis [25]. Overexpression of gene *CEP55* has been observed in variety of solid tumors, including colon cancer [26], bladder cancer [27], hepatocellular carcinoma [28], gastric cancer [29], esophagus adenocarcinoma [30] and ovarian carcinoma [24]. A previous study reported overexpression of CEP55 in 60% (9/15 samples) of CRC tissue samples [26]. Overexpression of *CEP55* activates p21 and enhances the cell cycle transition. In contrast, the knockdown of *CEP55* inhibits cell growth in gastric [29] and breast cancer [31]. Moreover, *CEP55* has an important role in final stage division, which involves the separation of two daughter cells [25, 32]. Overexpression of *CEP55* leads to an increase in the number of multinucleated cells and defect in cytokinesis, which may lead to tumorigenesis. In our set of genes, *FOXD3* and *FOXF2* have had a few studies performed on colon or gastric cancers. First, forkhead box D3 (FOXD3) was found to be a suppressor of colon cancer formation. While transcriptional repressor *FOXD3* is expressed in many types of embryonic cells, its knockdown dramatically increases human colon cancer cell proliferation, affecting the EGFR-Ras-Raf-MEK-ERK signaling pathway [33]. Methylation in the promoter region of another tumor suppressor *FOXF2* has previously been associated with shorter survival in gastric cancer patients. Through the FOXF2-IRF2BPL-β-catenin axis, *FOXF2* inhibits Wnt signaling by binding to E3 ligate *IRF2BPL* promoter and up-regulates *IRF2BPL*, which interacts with β-catenin for its ubiquitination and degradation [34]. Methylation of both tumor suppressors, *FOXD3* and *FOXF2*, could be responsible for their down-regulation, thus disturbing their interaction with other proteins.

The second set of three genes, *GNAO1*, *GRIA4* and *KCNA5*, has been less researched, with only a few studies related to cancer. Gene *GNAO1* was found to be overexpressed in 62.9% of patients with gastric cancer [35], while in our CRC tissue samples gene *GNAO1* was down-regulated. An association had been shown between overexpressed gene *GNAO1* and tumor size, tumor differentiation, TNM stage and poor prognosis. Their findings also demonstrated that knockdown of *GNAO1* leads to reduced proliferation and promotes the apoptosis of gastric cancer cells [35]. However, statistical evaluation of an effect of methylation status or expression of gene *GNAO1* on tumor size and TNM status in our case is impossible, since gene methylation was observed in the majority of samples. The second gene from this set, *GRIA4*, was the most methylated gene in our in silico study, with two probes being present in 98.4% of samples. Moreover, its down-regulation was experimentally confirmed in 98.1% of CRC tissue samples. A recently published study reported detecting a change in methylation in all CRC tissue samples, results similar to ours, and methylated cfDNA of *GRIA4* in 68.5% of metastatic CRC patients [36]. Potassium voltage-gated channel subfamily A member 5 (*KCNA5*) is a protein coding gene involved in tumor cell proliferation in Ewing sarcoma [37], while its role in CRC is still unknown. In our study, gene *KCNA5* was methylated in all studied samples, while its expression was decreased in 84% of our CRC tissue samples. Speculatively, as well as Ewing sarcoma, methylation of *KCNA5* could be responsible for stable silencing of this gene in CRC, thus contributing to proliferation of tumor cells.

We were not able to perform survival analysis, since the majority of samples had either a hypermethylated or hypomethylated promoter region of validated gene. Since expression analysis was performed on a small cohort of samples (*n* = 25), it did not seem reasonable to do survival analysis, e.g., for gene *FOXD3*, with which 68% of tumors had down-regulation of these gene and the remaining 32% had either no change or up-regulation.

A limitation of the study was that there were 115 samples available for experimental methylation analysis and only 25 for experimental expression analysis. There were also small discrepancies when comparing the methylation statuses of the entire cohort of 115 samples and 25 samples (Table 4). The biggest difference in methylation status was observed in the *FOXD3* gene, with which the discrepancy was 4%. Methylation status of *CEP55*, *FOXF2*, *GRIA4* and *KCNA5*, exhibited 2.7, 2.2, 1.3 and 0.9% discrepancy, while *GNAO1* was methylated in all samples, so showing no discrepancy.

## Conclusions

In summary, using bioinformatics on TCGA data followed by experimental validation we identified a set of six genes, *CEP55*, *FOXD3*, *FOXF2*, *GNAO1*, *GRIA4* and *KCNA5*, being differentially expressed in CRC compared to normal mucosa and whose expression seemed to be methylation dependent. The results of both approaches revealed that their change is frequent in CRC, regardless of their subtype, methylation clusters and the mutation status of CRC. As such, these six genes are believed to be an early event in human CRC carcinogenesis and to be potential CRC biomarkers.

## Additional files

Additional file 1: **Table S1.** Primers used for quantitative PCR experiment. (DOCX 15 kb)

Additional file 2: **Table S2.** Aberrantly methylated and differentially expressed genes. A list of probes aberrantly methylated belonging to differentially expressed genes in each cluster when compared to normal tissue samples. (DOCX 15 kb)

Additional file 3: **Table S3.** Aberrantly methylated and differentially expressed genes. A list of probes aberrantly methylated belonging to differentially expressed genes in each cluster when compared to normal tissue samples. (TIF 15 kb)

Additional file 4: **Figure S1.** Protein-protein interaction network (PPIN). The PPIN was performed using 198 differentially expressed genes from our study. (XLSX 372 kb)

Additional file 5: **Table S5.** Primers used for methylation-sensitive high resolution melting experiment. (XLSX 36 kb)

Additional file 6: **Table S6.** Literature mining analysis for differentially expressed genes from our study. (XLSX 10 kb)

## Abbreviations

COAD: Colon adenocarcinoma; READ: rectum adenocarcinoma
logFC: logarithm of fold change
MS-HRM: Methylation-sensitive high resolution melting
MSI: Microsatellite instable
MSI-H: Microsatellite instable-high
MSI-L: Microsatellite instable-low
MSS: Microsatellite stable
qPCR: Quantitative polymerase chain reaction
TCGA: The Cancer Genome Atlas; CRC: colorectal cancer
