# Supplementary material for: Genes CEP55, FOXD3, FOXF2, GNAO1, GRIA4, and KCNA5 as potential diagnostic biomarkers in colorectal cancer
Source: BMC Med Genomics. 2019 Apr 15;12:54. doi: 10.1186/s12920-019-0501-z (PMC6466812; doi:10.1186/s12920-019-0501-z)
Supplement: Supplementary file 1 — Table S1. Primers used for quantitative PCR experiment. (DOCX 15 kb) [file 12920_2019_501_MOESM1_ESM.docx]

| Gene | CpG |  | Sequence | Product length (bp) | Annealing temperature (°C) |
| --- | --- | --- | --- | --- | --- |
| *CEP55* | cg25314624 | F  R | 5’-TTTTTTTAAGTGAGATTTTATGTGG-3’  5’-CCATCCAATAAATAATACAAAACC-3’ | 99 | 57 |
| *FOXD3* | cg15617155 | F  R | 5’-GTCGGTTTTCGGTAGGA-3’  5’-TCCTACTAACCTTAACGCAAAA-3' | 81 | 52 |
| *FOXF2* | cg12221475 | F  R | 5’- GYGTTTATTAGGGTTGGAAGAA-3’  5’- CRCCCRAAACCCTTAAA-3’ | 85 | 52 |
| *GNAO1* | cg00866976  cg10273340 | F  R | 5’- TTTAAGGCGAATTTTGGG-3’  5’- CTCCAACCGCTAAAACC-3’ | 101 | 54 |
| *GRIA4* | cg00343633  cg03225817  cg07972135  cg23559689 | F  R | 5’-GTGTAGGTTTGTTGGGGG-3’  5’- ACTTTCTCACATACACACAAACACTAA-3’ | 90 | 58 |
| *KCNA5* | cg16897114 | F  R | 5’-GAAGAGAGAGAGGTAGAGAGTAGGGTA-3’  5’-ACGCCTAACGTCAAACTCC-3’ | 132 | 60 |
