# Supplementary material for: Genes CEP55, FOXD3, FOXF2, GNAO1, GRIA4, and KCNA5 as potential diagnostic biomarkers in colorectal cancer
Source: BMC Med Genomics. 2019 Apr 15;12:54. doi: 10.1186/s12920-019-0501-z (PMC6466812; doi:10.1186/s12920-019-0501-z)
Supplement: Supplementary file 2 — Table S2. Aberrantly methylated and differentially expressed genes. A list of probes aberrantly methylated belonging to differentially expressed genes in each cluster when compared to normal tissue samples. (DOCX 15 kb) [file 12920_2019_501_MOESM2_ESM.docx]

| Gene | Product length (bp) | Amplified exons | Detected transcripts | Name (Qiagen) |
| --- | --- | --- | --- | --- |
| *CEP55* | 135 | 3/4/5 | NM_001127182 (2534 bp)  NM_018131 (2656 bp)  XM_006717909 (2494 bp) | Hs_C10orf3_1_SG QuantiTect Primer Assay |
| *FOXD3* | 100 |  | NM_012183 (2078 bp) | Hs_FOXD3_1_SG QuantiTect Primer Assay |
| *FOXF2* | 102 |  | NM_001452 (2187 bp) | Hs_FOXF2_2_SG QuantiTect Primer Assay |
| *GNAO1* | 134 | 8/9 | NM_020988 (3332 bp) | Hs_GNAO1_1_SG QuantiTect Primer Assay |
| *GRIA4* | 119 | 12/13 | NM_000829 (5508 bp)  NM_001077243 (5621 bp)  XM_005271518 (5550 bp)  XM_006718823 (5180 bp) | Hs_GRIA4_1_SG QuantiTect Primer Assay |
| *KCNA5* | 113 |  | NM_002234 (2886 bp) | Hs_KCNA1_1_SG QuantiTect Primer Assay |
|  |  |  |  |  |
| *Endogenous controls* |  |  |  |  |
| *ACTB* | 146 | 3/4 | NM_001101 (1852 bp) | Hs_ACTB_1_SG QuantiTect Primer Assay |
| *GAPDH* | 112 | 2/3 | NM_002046 (1421 bp)  NM_001289745 (1513 bp)  NM_001289746 (1407 bp) | Hs_GAPDH_vb.1_SG QuantiTect Primer Assay |
| *RRN18S* | 149 |  | X03205 (1869 bp) | Hs_RRN18S_1_SG QuantiTect Primer Assay |
| *RPL13A* | 161 | 5/6/7 | NM_012423 (1196 bp) | Hs_RPL13A_1_SG QuantiTect Primer Assay |
