# Supplementary figures and images for: Genes CEP55, FOXD3, FOXF2, GNAO1, GRIA4, and KCNA5 as potential diagnostic biomarkers in colorectal cancer
Source: BMC Med Genomics. 2019 Apr 15;12:54. doi: 10.1186/s12920-019-0501-z (PMC6466812; doi:10.1186/s12920-019-0501-z)

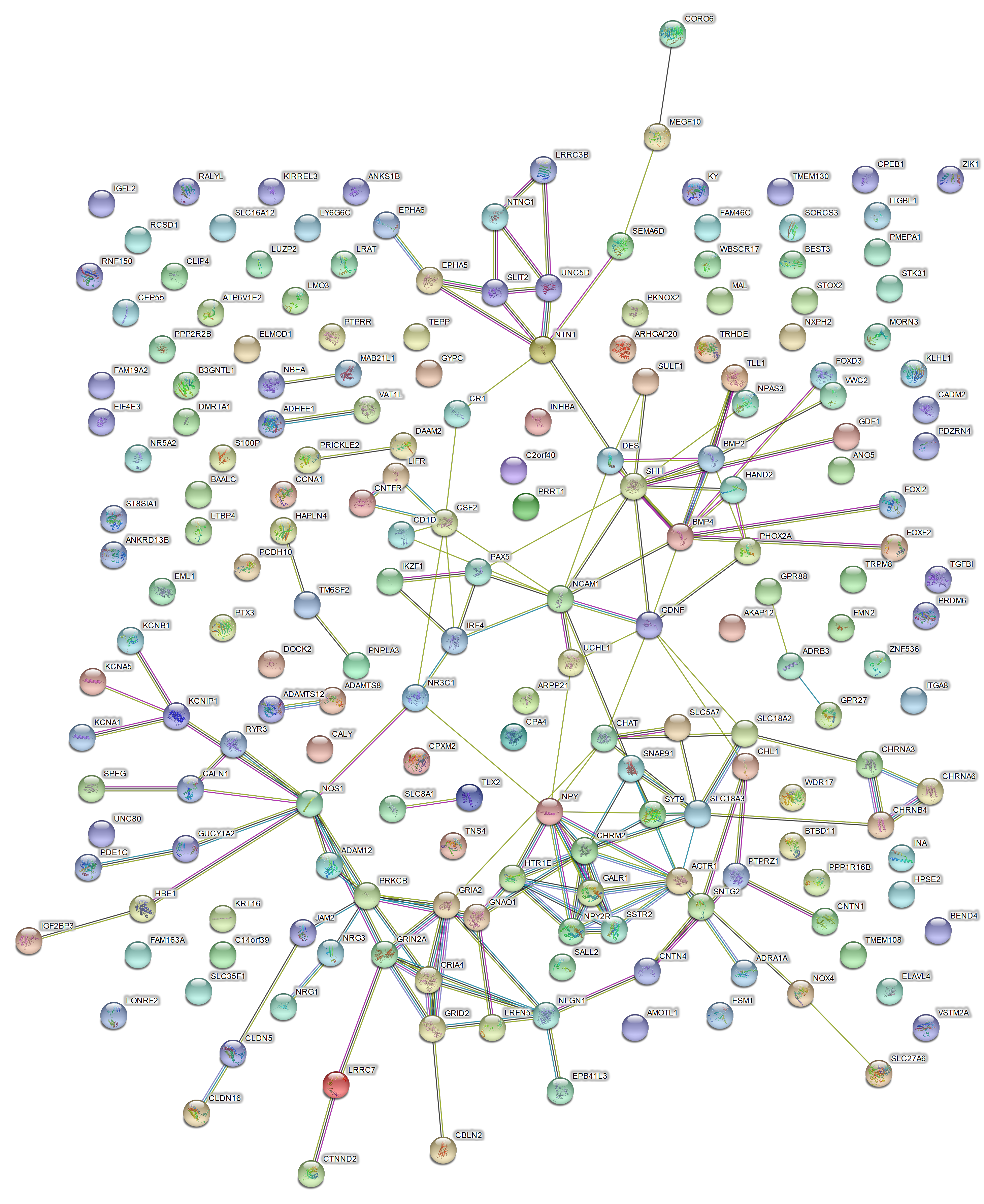

Supplement: Supplementary file 4 — Figure S1. Protein-protein interaction network (PPIN). The PPIN was performed using 198 differentially expressed genes from our study. (XLSX 372 kb) [file 12920_2019_501_MOESM4_ESM.tif]
